# Nutritional intervention in end-stage renal disease: a clinical trial study

**DOI:** 10.3389/fnut.2024.1322229

**Published:** 2024-10-24

**Authors:** Xueting Tao, Jiaolin Qian, Yongwei Hu

**Affiliations:** ^1^Department of Clinical Nutrition, The First People's Hospital of Taicang, The Affiliated Taicang Hospital of Soochow University, Suzhou, China; ^2^Department of Nephrology, The First People's Hospital of Taicang, The Affiliated Taicang Hospital of Soochow University, Suzhou, China

**Keywords:** end-stage renal disease, individualized nutritional interventions, diabetes mellitus, residual renal function, nephrology

## Abstract

**Objective:**

Chronic kidney disease is a global health problem, and end-stage renal disease (ESRD) has a major impact on patients' quality of life and prognoses. However, studies on individualized nutritional therapy for patients with ESRD need more complementary evidence.

**Methods:**

A clinical study was conducted based on a small population. It included patients with ESRD who underwent dialysis treatment in the Taicang Hospital Department of Nephrology, Soochow University, China, between January 2019 and December 2021. According to the randomized number table method, patients were divided into the nutritional treatment group (NIG) and the non-nutritional intervention control group (NNIG). There were 84 patients in the NIG and 92 patients in the NNIG. This study analyzed the changes in residual renal function (RRF) and indicators of blood and kidney function in ESRD with personalized nutritional therapy.

**Results:**

The results show that nutritional interventions for ESRD are effective in reducing the rate of decline in RRF and improving indicators of blood and kidney function in patients with ESRD. It was also found that patients with diabetes mellitus gained fewer health benefits per unit of RRF improvement with individualized nutritional therapy.

**Conclusion:**

This study provides important information about the treatment effects and factors associated with individual nutritional interventions in a population with ESRD. These results contribute to a better understanding of the effects of nutritional therapy in ESRD and provide a basis for managing it. Further studies should focus on specific populations and potential interventions to improve patient prognosis.

## 1 Introduction

End-stage renal disease (ESRD) is a serious kidney disorder characterized by the inability of the kidneys to function properly, leading to the accumulation of metabolic wastes and fluids in the body (1–3). The main symptoms of ESRD include oedema, hypertension, anemia and derangements in mineral and vitamin metabolism (4). If left untreated, ESRD can result in various complications such as cardiovascular diseases, bone disorders and infections, significantly affecting patients' quality of life and survival rates (5). The epidemiological trends of ESRD show an increasing global prevalence, with factors such as aging populations, increasing rates of chronic diseases like hypertension and diabetes mellitus (DM) and environmental pollution contributing to the rise (6). In the United States, the incidence of ESRD has increased from 362 per million population in 2000 to 489 per million population in 2016 (7). In China, the prevalence of ESRD has also demonstrated an upward trend. A nationwide survey-based study revealed that the incidence of ESRD increased from 103.6 per million population in 2009 to 129.4 per million population in 2015 (8, 9). The primary treatment for ESRD is renal replacement therapy, including haemodialysis, peritoneal dialysis and kidney transplantation, which help to remove waste products and excess fluids from the body (10). Pharmacological interventions are also employed to manage complications and target underlying causes. Additionally, lifestyle modifications such as dietary control, smoking cessation and appropriate exercise can help slow down the progression of kidney disease (11).

The importance of food and nutrition for health has long been appreciated, and attempts have been made to prevent and treat disease through dietary modifications (12). In modern medicine, significant progress has been made in the study of nutritional interventions for the treatment of disease (13). Researchers have found that nutrients and dietary components can have profound effects on physiological functioning, thereby influencing the development and outcome of chronic kidney disease (14). Nutritional interventions to treat disease include dietary modification, nutritional supplementation and pharmacological nutritional therapy (15). Due to impaired kidney function, patients with ESRD are prone to malnutrition and mineral and vitamin metabolism disorders (16). Through nutritional intervention therapy, patients can maintain good nutritional status, reduce the burden on the kidneys and lower the risk of complications (17). Furthermore, researchers have found that a low-protein diet can help delay the progression of chronic kidney disease (18). In addition, vitamin D, calcium and phosphorus mineral supplements are vital to maintaining bone health in patients with ESRD (19). In terms of pharmaco-nutritional therapy, nutrients such as omega-3 fatty acids and vitamin C have also been shown to protect the kidneys and reduce inflammatory responses (20).

Nutritional intervention therapy has an important role in the treatment of ESRD, and it has been reported to help patients improve their nutritional status, slow down disease progression and improve their quality of life by adjusting their diets and supplementing them with nutrients (21–23). The relationship between nutritional interventions and blood- and kidney-related indicators has not been thoroughly explored in other studies. Therefore, this study analyses the role of nutritional therapy in the treatment of residual renal function (RRF), as well as the relationship between the organism's blood and kidney indicators and nutritional therapy. Since the needs and responses to nutrients vary among patients, an individualized nutritional treatment plan is needed to optimize the therapeutic effect (24). This study included patients with ESRD undergoing dialysis treatment. It intervened in their diets through personalized nutritional interventions, observed the effects of personalized nutritional therapy and noted the impact of DM in nutritional intervention for ESRD.

## 2 Materials and methods

### 2.1 Participants

This study included patients with ESRD who underwent dialysis treatment in the Taicang Hospital Department of Nephrology, Soochow University, between January 2019 and December 2021. According to the consensus of the International Society of Nephrology and the International Diabetes Federation, a glomerular filtration rate of < 15 mL/min/1.73m^2^ is sufficient to diagnose ESRD.

**Figure 1:**
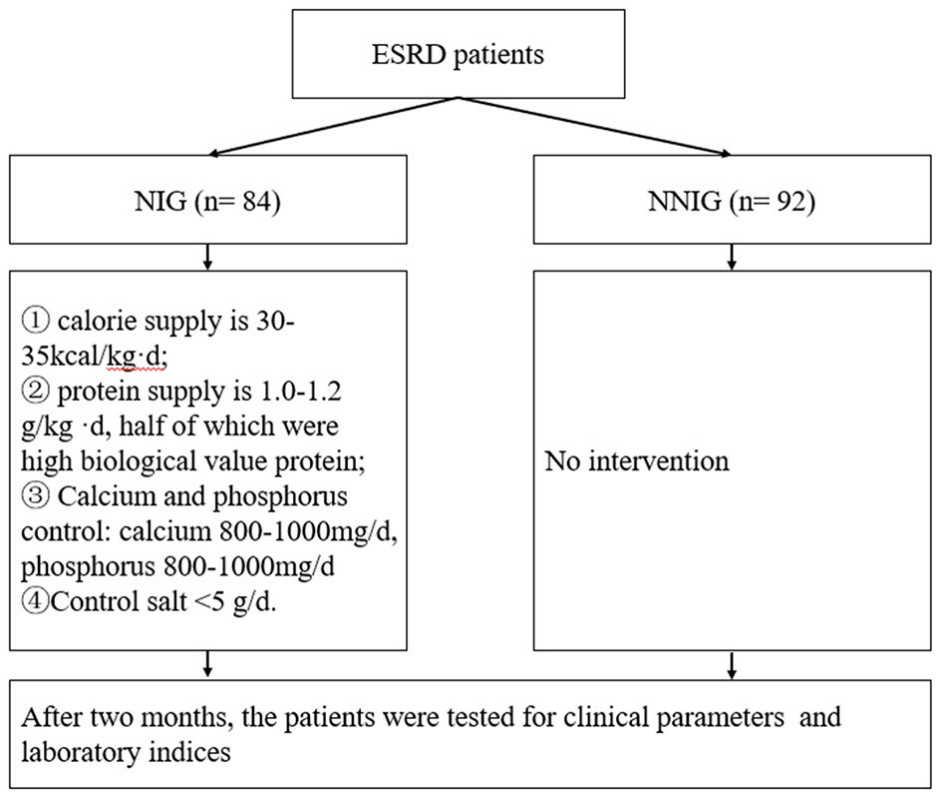
Flowchart of this study design.

According to the randomized number table method, patients were divided into the nutritional treatment group (NIG) and the non-nutritional intervention control group (NNIG). There were 84 patients in the NIG and 92 patients in the NNIG as shown in Figure 1. The inclusion criteria were as follows: 1. aged over 18 years; 2. haemodialysis treatment for at least 60 days; 3. provided signed informed consent. The exclusion criteria were as follows: 1. suffering from acute kidney injury; 2. pre-dialysis residual glomerular filtration rate < 2 ml/(min-1.73m^2^) or urine volume (UV) < 500 ml/24 h; 3. suffering from liver disease, active tuberculosis, malignant tumors or serious infections; 4. did not provide signed informed consent. This study was approved by the Ethics Committee of Taicang First People's Hospital. Moreover, the researchers explained in detail the purpose, methodology, potential risks and benefits of this study to the patients and ensured that their informed consent for participating in the research was obtained.

A disposable dialyser was used, the membrane material was polysulfone with an area of 1.5–1.7 m^2^. A standard sodium bicarbonate dialysis solution was used, the dialysate flow rate was 500 ml/min, the blood flow rate was 200–300 ml/min and the dialysis frequency was 3 times/week at 4 h/session. Ordinary heparin or low-molecular heparin was used for anticoagulation.

### 2.2 Measures of intervention

In this study, patients in the NIG were treated with nutritional interventions. (1) Nutritional assessment was performed according to the Subjective Global Assessment and the Biochemical Components Analysis. (2) Nutritional guidance was provided with the help of food modeling and using the standard portion size of food in the Chinese Dietary Guidelines for Chinese Residents. Nutritional guidance: the patients' dietary intake was collected using the 3-day, 24-h dietary review method (including 2 working days and 1 rest day) as a reference. An individualized nutritional intervention plan was formulated according to the recommendations of the Guidelines on the Prognostic Quality of Kidney Diseases. The applied principles of nutritional treatment were as follows: 1. calorie supply is 30–35 kcal/kg per day; 2. protein supply is 1.0–1.2 g/kg per day, half of which were high biological value protein; 3. calcium and phosphorus control: calcium 800–1000mg/day, phosphorus 800–1,000 mg/day; 4. controlled salt intake < 5 g/d; 5. nutritional follow-up: the patient should correctly record their daily diet (including the name of the food, raw materials, weight, time and form of cooking), and the patient should be followed-up every 2 weeks. Patients in the NNIG group received only conventional dialysis treatment. The patients' daily diet was recorded, and the results are detailed in Supplementary Table 1.

### 2.3 Data collection

After 2 months of treatment, patients' clinical parameters were collected, including demographic indicators (age, sex, body mass index [BMI], primary disease) and laboratory indices (mean arterial pressure [MAP], ultrafiltration volume [UAV], hemoglobin [Hb], albumin [Alb], blood calcium [Ca], blood phosphorus [P], parathyroid hormone [PTH], alkaline phosphatase [ALP] and high-sensitive C-reactive protein, N-terminal prohormone of brain natriuretic peptide [BNP] and urea clearance index [Kt/V]). In this study, RRF was defined as the rate of decline of the glomerular filtration rate of the residual kidney. If the patient's non-dialysis daily UV >100 ml, they were considered to have RRF, which was calculated as:


RFF= (Cu×V)/(C. + C1)/2)×t

where Cu is urinary urea (mol/L), V is the 24-h UV (m1), C. is the post-dialysis urea (mmol/L), C1 is the pre-dialysis urea (mmol/L) and t is the interval between dialysis (min). The series values of patients' RRF were analyzed at each follow-up visit using linear regression with time (months), and the slope of the linear regression was the RRF.

### 2.4 Quality control and assessment

The study followed the Consolidated Standards of Reporting Trials statement and the Strengthening the Reporting of Observational Studies in Epidemiology guidelines to ensure the reliability and validity of the research methodology. The quality of the data was regularly assessed by the researchers to ensure its accuracy and completeness.

### 2.5 Statistical analysis

The SPSS 26.0 software was used for statistical analysis, and the independent samples *t*-test or χ2 test was used for group comparisons. For normally distributed measurement data, the mean ± standard deviation (*x* ± *s*) was used. The Pearson correlation analysis was conducted using the corrplot package in R to explore the relationship between the indicators. Simple linear regression was used to explore the linear relationship between the indicators, and a value of *P* < 0.05 was considered statistically significant.

## 3 Results

### 3.1 General information on the participants

A total of 176 patients with ESDR were included in this study comprising 84 patients in the NIG group and 92 in the NNIG group. There was no significant difference between the two groups in terms of sex (43 men/41 women for the NIG; 53 men/39 women for the NNIG), age (62.2 ± 9.9 for the NIG; 64.8 ± 10.5 for the NNIG) and BMI (23.1 ± 2.7 for the NIG; 23.1 ± 2.4 for the NNIG) (*P* > 0.05). A significant difference was, however, found in the presence of DM prior to admission (11.9% for the NIG and 27.2% for the NNIG) between the two groups (*P* = 0.002). Therefore, in subsequent analyses, patients in the NIG and NNIG were separated by diabetic stratification. Nutritional interventions and DM had little effect on Ca levels (see Table 1).

**Table 1:** General information about the patient.

	**NIG**	**NNIG**	***P* value**
Sex (male/female)	43/41	53/39	0.158^#^
Age (mean ± SD)	62.2 ± 9.9	64.8 ± 10.5	0.097^*^
BMI (mean ± SD)	23.1 ± 2.7	23.1 ± 2.4	0.976^*^
DM (yes/no)	10/74	25/67	**0.002** ^**#**^

NIG, Nutrition intervention group; NNIG, Non-nutrition intervention group; SD, standard deviation; BMI, Body Mass Index; DM, diabetes mellitus. ^#^P-values obtained by using the chi-square test. ^*^P-values obtained by using the student's t test.

### 3.2 Improvement of nutritional interventions on patients' blood indicators

In patients with ESDR, blood calcium, blood phosphorus, albumin, UAV, RFF, and Kt/V responded to patients' health indicators and renal function. Compared with NNIG patients, there was a significant improvement in blood phosphorus, ALB, UAV, and RFF in the NIG group, regardless of whether the patients had diabetes or not. In the diabetic group, the nutritional intervention significantly reduced the Kt/V values, which were not found in the NIG. In the diabetic group, the nutritional intervention significantly reduced the Kt/V values, which was not found in the NIG. but the Kt/V values of all patients were within the health range. In the NNIG, there was no significant difference between the blood indices of the population with diabetes mellitus and non-diabetic population. However, the RFF of diabetic patients was significantly lower than that of non-diabetic population in NIG (see Table 2).

**Table 2:** Effect of nutritional interventions on renal function in patients.

		**NIG**	**NNIG**	***P* value**
Ca (nmol/L)	DM	2.03 ± 0.26	1.98 ± 0.2	0.610
	Non-DM	2.04 ± 0.25	2.03 ± 0.27	0.778
	*P* value	0.961	0.279	
*P* (nmol/L)	DM	1.77 ± 0.22	2.28 ± 0.48	**< 0.001**
	Non-DM	2.27 ± 0.43	1.79 ± 0.35	**< 0.001**
	*P* value	0.905	0.912	
Alb (g/L)	DM	33.95 ± 3.33	28.6 ± 2.59	**< 0.001**
	Non-DM	35.32 ± 3.54	29.41 ± 2.72	**< 0.001**
	*P* value	0.257	0.206	
UAV (ml/kg/h)	DM	3.15 ± 0.57	4.04 ± 1.03	**< 0.001**
	Non-DM	3.32 ± 0.96	4 ± 1.02	**< 0.001**
	*P* value	0.597	0.853	
RFF	DM	−3.39 ± 1.39	−4.74 ± 1.29	**< 0.001**
	Non-DM	−2.45 ± 1.03	−4.57 ± 1.17	**< 0.001**
	*P* value	**0.012**	0.56	
Kt/V	DM	1.25 ± 0.05	1.33 ± 0.11	**< 0.001**
	Non-DM	1.29 ± 0.06	1.29 ± 0.08	0.488
	*P* value	**0.034**	0.06	

NIG, Nutrition intervention group; NNIG, Non-nutrition intervention group; DM, diabetes mellitus; Alb, albumin; UFV, ultrafiltration volume; RRF, residual renal function; Kt/V, dialysis effectiveness index. All P-values obtained by using the student's t test. Significance level symbol: *P* value is less than 0.05, the result has statistical significance.

### 3.3 Impact of nutritional interventions on indicators of blood and kidney function

Nutritional interventions also had an impact on patients' blood and renal function indices. The results indicate that nutritional interventions also significantly reduce Alp levels and increase Hb concentrations, regardless of whether patients have DM or not. In addition, nutritional interventions significantly reduced BNP levels in patients without DM; however, in patients without DM, a decrease in BNP levels could not be induced. This study revealed that nutritional interventions did not affect PTH, MAP, TG and TC in patients with or without DM (see Table 3).

**Table 3:** Effect of nutritional interventions on other indicators.

		**NIG**	**NNIG**	***P* value**
iPTH (pg/ml)	DM	215.71 ± 90.98	234.21 ± 92.8	0.606
	Non-DM	205.8 ± 95.5	238.57 ± 116.04	0.070
	*P* value	0.76	0.868	
Alp (U/L)	DM	94.42 ± 27.69	154.65 ± 62.49	**0.007**
	Non-DM	101.43 ± 40.21	147.04 ± 62.07	**< 0.001**
	*P* value	0.599	0.607	
BNP (pg/ml)	DM	7262.34 ± 2302.22	8207.9 ± 1477.09	0.171
	Non-DM	5518.38 ± 2059.09	8331.04 ± 1874.35	**< 0.001**
	*P* value	**0.017**	0.77	
HB (g/L)	DM	98.7 ± 11.24	81.48 ± 11.43	**< 0.001**
	Non-DM	99.12 ± 11.87	79.16 ± 12.77	**< 0.001**
	*P* value	0.917	0.433	
MAP (mmgh)	DM	95.2 ± 12.91	90.2 ± 12.28	0.305
	Non-DM	93.92 ± 12.35	91.87 ± 13.86	0.357
	*P* value	0.763	0.602	
TG (mmol/L)	DM	1.84 ± 0.12	1.87 ± 0.1	0.413
	Non-DM	1.92 ± 0.45	1.9 ± 0.25	0.719
	*P* value	0.574	0.643	
TC (mmol/L)	DM	4.33 ± 0.81	4.39 ± 0.8	0.842
	Non-DM	4.41 ± 0.86	4.48 ± 0.86	0.617
	*P* value	0.784	0.649	

DM, diabetes mellitus; iPTH, Intact Parathyroid Hormone; Alp, Alkaline Phosphatase; BNP, Brain Natriuretic Peptide; HB, hemoglobin; MAP, mean arterial pressure; TG, triglyceride; TC, total cholesterol. All P-values obtained by using the student's t test. Significance level symbol: *P* value is less than 0.05, the result has statistical significance.

### 3.4 The impact of diabetes mellitus and residual renal function on patient health

**Figure 2:**
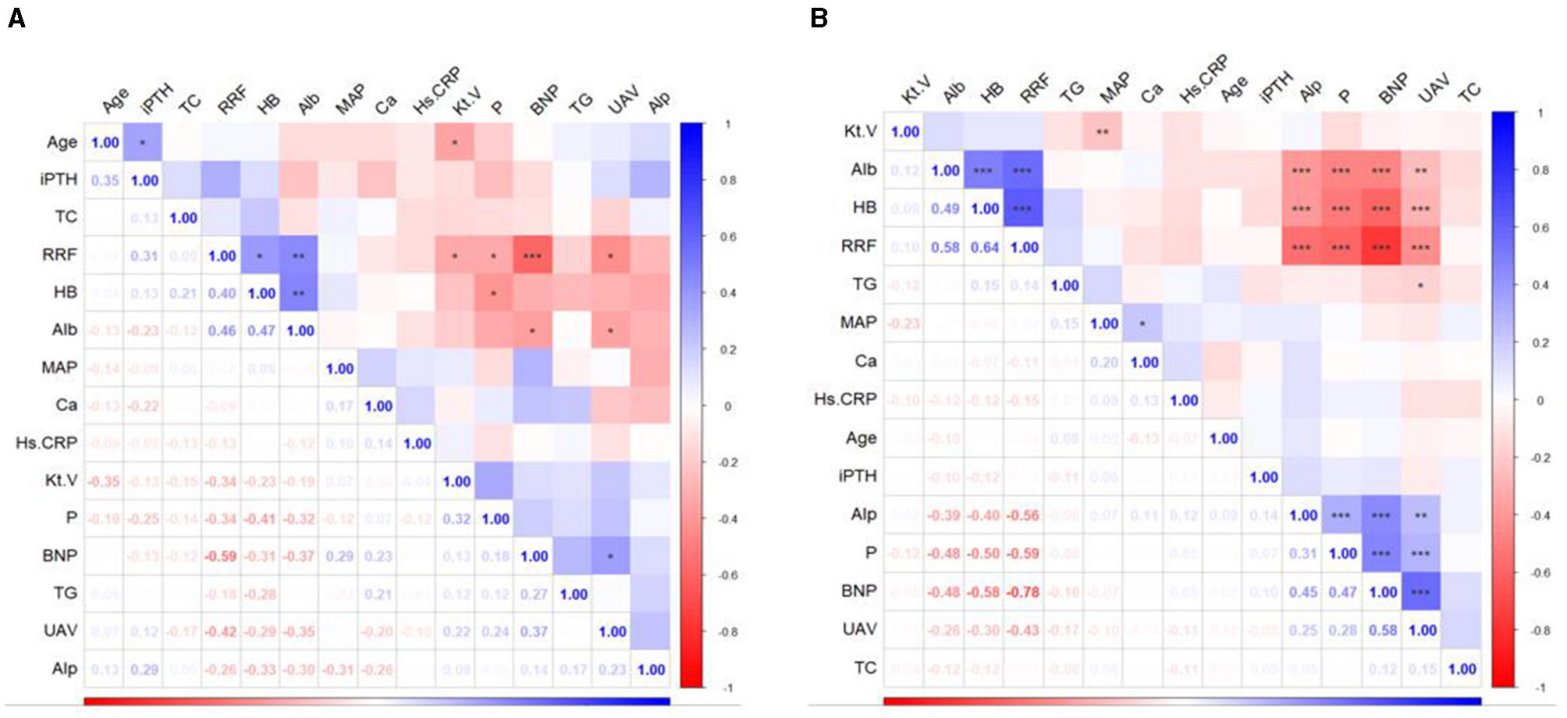
Heatmap of the correlations between indicators in the participants. **(A)** Patients with DM; **(B)** patients without DM. DM, diabetes mellitus; iPTH, Intact Parathyroid Hormone; TC, total cholesterol; RRF, residual renal function; HB, hemoglobin; Alb, albumin; MAP, mean arterial pressure; Ca, calcium; Hs.CRP, high sensitivity C-reactive protein; Kt.V, urea clearance index; P, phosphorus; BNP; brain natriuretic peptide; TG, triglyceride; UAV, ultrafiltration volume; Alp, alkaline phosphatase.

**Figure 3:**
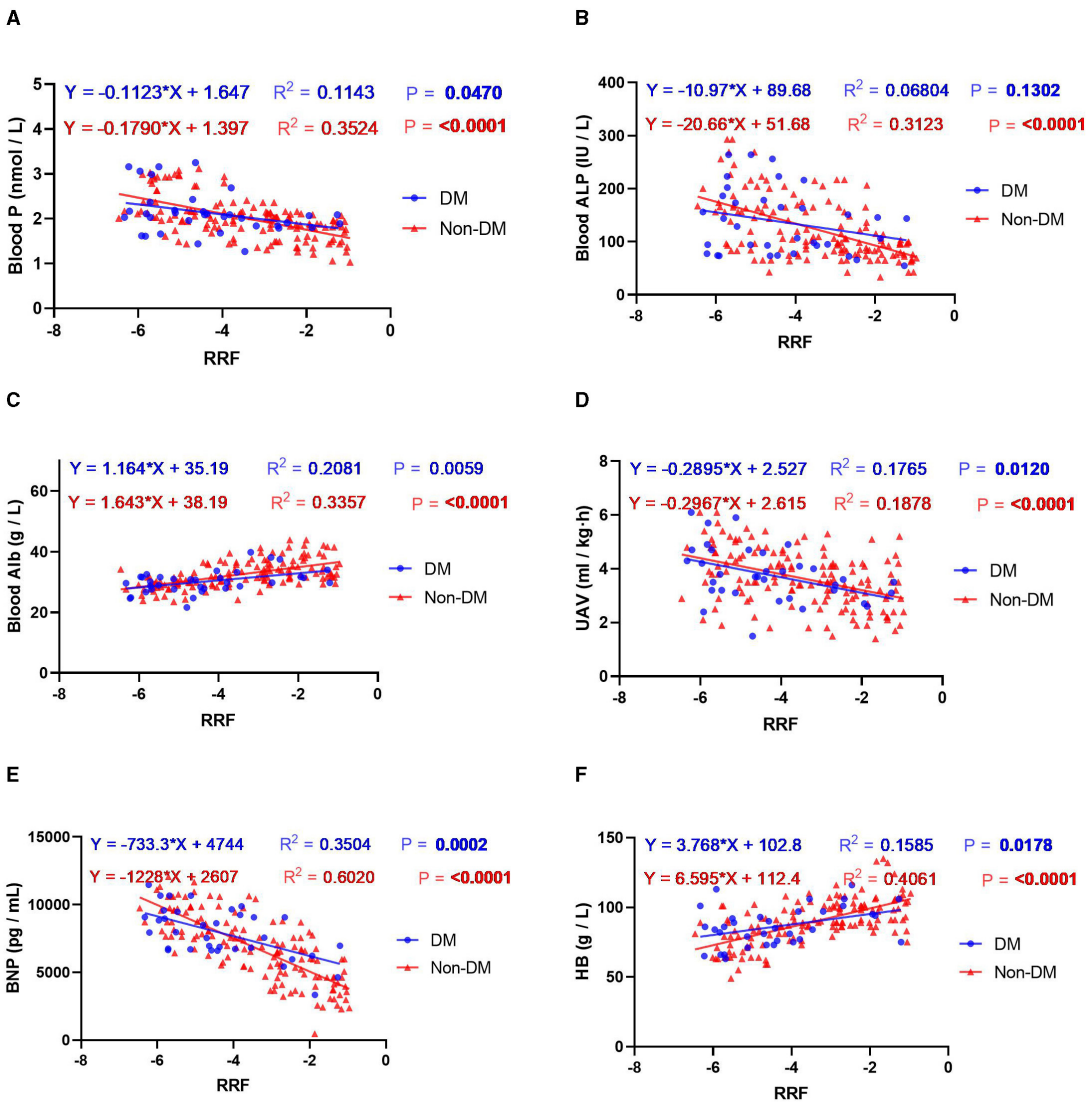
Regression curve analysis of the correlation between RRF and the indicators of blood and kidney function: **(A)** blood P; **(B)** blood ALP; **(C)** blood Alb; **(D)** UAV; **(E)** BNP; **(F)** HB. Blue indicates the presence of DM and red denotes no DM.

In addition to exploring the relationship between nutritional interventions and patients' blood and kidney functioning, this study analyzed the relationship between the rate of decline in RRF and indicators of blood and kidney function in patients with or without DM. The results showed that the rate of RRF decline in patients with DM was mainly related to the levels of HB, Alb, Kt/V, P, BNP and UAV. The rate of decrease in RRF in patients without DM was mainly related to Alb, HB, Alp, P, BNP and UAV (Figure 2). Further linear regression showed that each one-unit improvement in the rate of RRF decline had a significant effect on P-levels (0.1 mmol/l lower for patients with DM, and 0.2 mmol/l lower for patients without DM), Alp levels (11.0 IU/L lower for patients with DM, and 20.7 IU/L lower for patients without DM), UAV levels (0.29 ml/kg/h lower for patients with DM, and 0.3 ml/kg/h lower for patients without DM), BNP levels (733.3 pg/ml lower for patients with DM, and 1,228 pg/ml lower for patients without DM), Alb levels (1.2 g/l higher for patients with DM, and 1.6 g/ml higher for patients without DM), HB levels (lower for patients without DM), HB levels (3.8 g/l higher for patients with DM, and 6.6 g/ml higher for patients without DM) as shown in Figure 3. Improvements per unit of RFF in patients with DM only resulted in fewer decreases in P (61.1%), ALP (54.4%), UAV (97.6%) and BNP (59.7%). as well as fewer rises in Alb (70.8%) and HB (57.1%) than in patients without DM. These results suggest that the rate of decline in RFF in patients with or without DM is not consistently related to indicators of blood and renal functioning. Differentiation between diabetic and nondiabetic patients is needed when using these metrics to predict RFF.

## 4 Discussion

End-stage renal disease is a severe kidney failure condition for which patients often require dialysis or kidney transplantation. At this stage, patients' nutritional status is often severely affected, including inadequate protein intake, increased energy expenditure and electrolyte imbalances. Traditional nutritional therapy is usually based on general guidelines, which overlook the existence of individual differences among patients. Individualized nutrition therapy emphasizes the importance of individual differences and provides a method for nutritional intervention based on patient characteristics and needs. This study included 176 patients with ESRD and observed the impact of individualized nutrition therapy on various indicators of the body. It was found that individualized nutrition therapy effectively reduced the rate of decline in residual kidney function, improved kidney function indicators and enhanced overall nutritional status. Furthermore, the effectiveness of individualized nutrition therapy in patients with DM was inferior to that in patients without DM, suggesting the need for further research to explore personalized nutrition therapy for patients with DM. This study's findings provide important guidance for clinical practice and contribute to improving treatment outcomes and quality of life for patients with ESRD.

Individualized nutrition therapy is an important component of ESRD treatment (12, 25). It not only improves the symptoms and complications of uraemia, such as electrolyte acid–base imbalance, water and salt metabolism disorders and mineral and bone metabolism abnormalities but also slows down the decline of renal function (26–28). Dietary management methods for ESRD mainly include restricting protein intake, controlling sodium intake, managing fluid intake and supplementing vitamins and minerals (29). Among these, protein restriction is at the core of dietary management for ESRD (30). Moderate restriction of protein intake can reduce the burden on the kidneys and delay disease progression. Additionally, appropriate protein restriction can reduce the incidence of uraemia and the risk of death (31). Furthermore, patients with ESRD often have reduced urine output or polyuria, which can easily lead to fluid retention (32). Therefore, sodium intake should be limited, and it is generally recommended that no more than 2–3 g of sodium be consumed per day while controlling fluid intake (33). In addition, supplementing vitamins and minerals such as vitamin B complex, vitamin C, calcium and iron can help improve the nutritional status of patients (34). In this study, individualized nutritional therapy significantly delayed the progression of ESRD and improved the health status of patients.

Diabetic nephropathy is the main cause of ESRD and one of the most common complications of DM (35, 36). Prolonged hyperglycaemia damages the micro-vessels and glomeruli of the kidneys, leading to a gradual decline in renal function (37, 38). It usually develops after many years in patients with DM and manifests itself as proteinuria, hypertension and a gradual decline in renal function (39). This study found that individualized nutritional therapy was less beneficial for patients with ESRD and DM than for patients with ESRD but not DM. Moreover, there were differences between patients with and without DM in terms of the association of RRF decline rates with blood and renal function indices. This was manifested as a trend loss in BNP improvement and fewer health gains from a lower rate of deterioration in unit renal function. Therefore, for patients with ESRD combined with DM, a comprehensive nutritional intervention programme for DM may be needed to further improve the effectiveness of individualized nutritional therapy.

In summary, individualized nutritional therapy can improve the therapeutic efficacy and the organic status of patients with ESRD by controlling or supplementing the intake of appropriate substances and improving the metabolism of nutrients.

This study has some limitations. It was a single-center study based on a small population, and the time of intervention and observation was relatively short. A multicentre, long-term and more in-depth exploration of a larger population is needed to prove the efficacy of nutritional therapy and provide indications for making timely adjustments to develop a more thorough therapy programme for patients with different disease progressions.

## Data Availability

The raw data supporting the conclusions of this article will be made available by the authors, without undue reservation.
