# Supplementary material for: Nutritional intervention in end-stage renal disease: a clinical trial study
Source: Front Nutr. 2024 Oct 24;11:1322229. doi: 10.3389/fnut.2024.1322229 (PMC11541048; doi:10.3389/fnut.2024.1322229)
Supplement: Supplementary file 1 [file Table_1.DOCX]

Taicang First People's Hospital

24-hour dietary records

| name | Data |  |  | Days in group |  |
| --- | --- | --- | --- | --- | --- |
|  | Food name | food material | Quantity (g/mL) | Dining Location | note |
| breakfasts |  |  |  |  |  |
|  |  |  |  |  |  |
|  |  |  |  |  |  |
|  |  |  |  |  |  |
|  |  |  |  |  |  |
| additional food |  |  |  |  |  |
|  |  |  |  |  |  |
| lunch |  |  |  |  |  |
|  |  |  |  |  |  |
|  |  |  |  |  |  |
|  |  |  |  |  |  |
|  |  |  |  |  |  |
|  |  |  |  |  |  |
| additional food |  |  |  |  |  |
|  |  |  |  |  |  |
| dinner |  |  |  |  |  |
|  |  |  |  |  |  |
|  |  |  |  |  |  |
|  |  |  |  |  |  |
|  |  |  |  |  |  |
|  |  |  |  |  |  |
| additional food |  |  |  |  |  |
|  |  |  |  |  |  |

Note: It is important to keep track of food eaten throughout the 24 hours of the day, including three meals a day, additional meals (fruit, bread, etc.), water, oil, salt, seasonings used for cooking, and snacks (including beverages, candies, melons, chocolates, cookies, etc.)
